# Mixing Improvement in a T-Shaped Micro-Junction through Small Rectangular Cavities

**DOI:** 10.3390/mi13020159

**Published:** 2022-01-21

**Authors:** Matteo Antognoli, Sara Tomasi Masoni, Alessandro Mariotti, Roberto Mauri, Maria Vittoria Salvetti, Elisabetta Brunazzi, Chiara Galletti

**Affiliations:** Dipartimento di Ingegneria Civile e Industriale, Università di Pisa, Largo Lazzarino 2, 56122 Pisa, Italy; matteo.antognoli@phd.unipi.it (M.A.); sara.tomasimasoni@phd.unipi.it (S.T.M.); alessandro.mariotti@unipi.it (A.M.); roberto.mauri@unipi.it (R.M.); mv.salvetti@ing.unipi.it (M.V.S.); elisabetta.brunazzi@unipi.it (E.B.)

**Keywords:** T-shaped micro-junction, small rectangular cavities, flow regimes, mixing degree, numerical simulations

## Abstract

The T-shaped micro-junction is among the most used geometry in microfluidic applications, and many design modifications of the channel walls have been proposed to enhance mixing. In this work, we investigate through numerical simulations the introduction of one pair of small rectangular cavities in the lateral walls of the mixing channel just downstream of the confluence region. The aim is to preserve the simple geometry that has contributed to spread the practical use of the T-shaped micro-junction while suggesting a modification that should, in principle, work jointly with the vortical structures present in the mixing channel, further enhancing their efficiency in mixing without significant additional pressure drops. The performance is analyzed in the different flow regimes occurring by increasing the Reynolds number. The cavities are effective in the two highly-mixed flow regimes, viz., the steady engulfment and the periodic asymmetric regimes. This presence does not interfere with the formation of the vortical structures that promote mixing by convection in these two regimes, but it further enhances the mixing of the inlet streams in the near-wall region of the mixing channel without any additional cost, leading to better performance than the classical configuration.

## 1. Introduction

Micromixers—constituted by channels having a hydraulic diameter below or equal to 1 mm—have been widely proposed in the past years for liquid mixing because they allow continuous operation with exceptional control over transport phenomena and residence time. Working in continuous flow reduces the accumulation of reactive or toxic intermediates, and the high surface-to-volume ratio provides enhanced heat transfer capacity granting safer operation with highly exothermic reactions, thus expanding the number of feasible reactions that can be performed and intensified [1,2,3,4,5]. Microfluidic devices have been proposed in addition to labs-on-chips [6] as they lead to economic, environmental, and safety benefits for several processes in pharmaceutical and fine-chemistry fields [7,8,9]. One of the main disadvantages in the use of micromixers is that liquid mixing has to take place in laminar flow regimes for typical low Reynolds numbers, thus mixing needs to be enhanced by exploiting active or passive methods [10,11,12,13,14]. The active methods apply outsourced energy, such as ultrasound, pressure pulse, and electric and magnetic fields [15,16,17,18,19]. The passive ones, instead, promote mixing by devising a clever geometry of the micromixers aimed at breaking the flow symmetries without any external energy input.

In this context, the simplest and most studied configuration is the planar T-shaped micro-junction. As the Reynolds number varies, different flow regimes and the related mixing performance are well characterized in the literature [20,21,22,23,24,25,26,27,28,29,30,31,32,33]. In particular, the T-geometry exhibits a segregated regime at low Reynolds number. Then, by increasing the Reynolds number, a breaking of the flow symmetry occurs resulting in an improvement of mixing through convection. Many investigations have been aimed at understanding how this engulfment regime is affected by the cross-sectional aspect ratio [34,35] and geometry [36,37], and at characterizing the interplay between mixing performance and the reaction yield [38,39,40,41,42,43,44].

Among passive methods to enhance mixing, several modifications of the planar T-shaped micro-junction have been proposed. Most of them may be summarized in three main strategies: (i) the change of the angle between the inlet channels and the mixing channel to anticipate the onset of the engulfment regime number at a lower Reynolds number or keeping a T-geometry with not aligned inlet channels, (ii) the introduction of obstacles to force the development of asymmetric flow regimes, and (iii) the use of curved lateral walls to promote mixing thanks to recirculations.

As for the first strategy, arrow-shaped junctions, in which the axis of the inlet channels is tilted downward with respect to the mixing channel, allow us to achieve the onset of the engulfment regime at significantly lower Reynolds numbers compared to T-mixers. However, the mixing performance does not monotonically increase with Reynolds [45]. On the contrary, the onset of the engulfment regime occurs at a larger Reynolds number if Y-shaped junctions are used and, thus, this device appears to be less efficient in mixing [46,47,48]. Not-aligned inlet channels are proposed, in [49,50,51], to promote the formation of a central vortex in the outlet channel, which enhances mixing.

Zhang et al. [52] introduce a cylindrical pin in the mixing channel to force the development of asymmetric flow regimes through the perturbation of the interface between the two inlet streams. From this idea, more complex sequences of obstacles are derived and optimized for flow-symmetry breaking [53,54,55,56,57,58,59,60,61].

Finally, the use of sinusoidal walls in the mixing channel, instead of straight ones, is employed to promote mixing thanks to near-wall recirculations. Recirculations along the mixing channel can reduce the mixing length [62,63,64,65,66,67,68,69]. The drawback is that this latter strategy requires a radical and complex modification of the mixing channel compared to the previous strategies that leads to a more challenging micro-fabrication. Nevertheless, single or multiple recirculations, passively formed inside sinusoidal or suitably-shaped cavities, have been already proposed as a flow control device in many applications in internal and external flows. Small cavities are usually introduced in the diverging curved wall of diffusers in [70,71] to delay flow separation for the so-called “roller bearing” mechanism, i.e., the formation of a succession of recirculation regions adjacent to the solid surface. Single and multiple contoured transverse cavities were successfully introduced in the diverging walls of plane diffusers to delay or, if possible, to avoid flow separation, thus increasing the pressure recovery both in laminar [72] and in turbulent conditions [73]. Furthermore, recirculating flows in rectangular micro-cavities have been recently employed for clinical applications such as reaction chambers in miniaturized diagnostic devices [74]. Indeed, the flow induced by cavities can provide excellent size-based separation of particles or cells [75,76,77,78]. Interestingly, under specific conditions, fluid streamlines may enter and exit the cavity recirculation at different locations of the main channel flow, so they can potentially enhance mass and heat transfer in microdevices [79,80].

We are interested in evaluating the effect of a local modification of the lateral walls of the mixing channel, consisting of the introduction of one pair of small rectangular cavities just downstream of the confluence region. Compared with the strategy of modifying the whole microchannel (as in [63,67,68,69]), we want to see if only one local modification of the mixing channel is effective in improving mixing; this represents a challenging task. Thereby, we want to preserve the simple geometry that has contributed to spreading the use of the T-shaped micro-junction in many applications. This modification should, in principle, work jointly with the vortical structures present in the mixing channel, further enhancing their efficiency in mixing without significant additional pressure drops.

In this paper, the performance of the passive flow control device is numerically evaluated in the different flow regimes occurring in the microdevice by increasing the Reynolds number and using the same inflow rates. In particular, in Section 3.1 for the selected Reynolds numbers, we compared the obtained results between the T-shaped micro-junction (TJ) and the same geometry with cavities (called herein T-shaped micro-junction with Cavities, TJC). We carried out a post-process analysis to gain insight into how the fluid motion affects the mixing mechanism in the presence of cavities in Section 3.2. Indeed, this investigation gives insight into the mechanism triggering mixing with cavities, and, in particular, into the interplay between the recirculation embedded in the cavity region and the vortical structures formed in the different flow regimes of the T-shaped micro-junction. To our knowledge, these aspects have not been investigated in the literature to date. We also provide a robustness analysis in Section 3.3 to understand how the cavity size affects the mixing efficiency, from which practical indications for operative limits and the fabrication process of the device can be derived. Hence, the introduction of small cavities may represent an applicable and affordable modification in the design of the TJ.

## 2. Numerical Model

### 2.1. Geometry and Parameters

**Figure 1:**
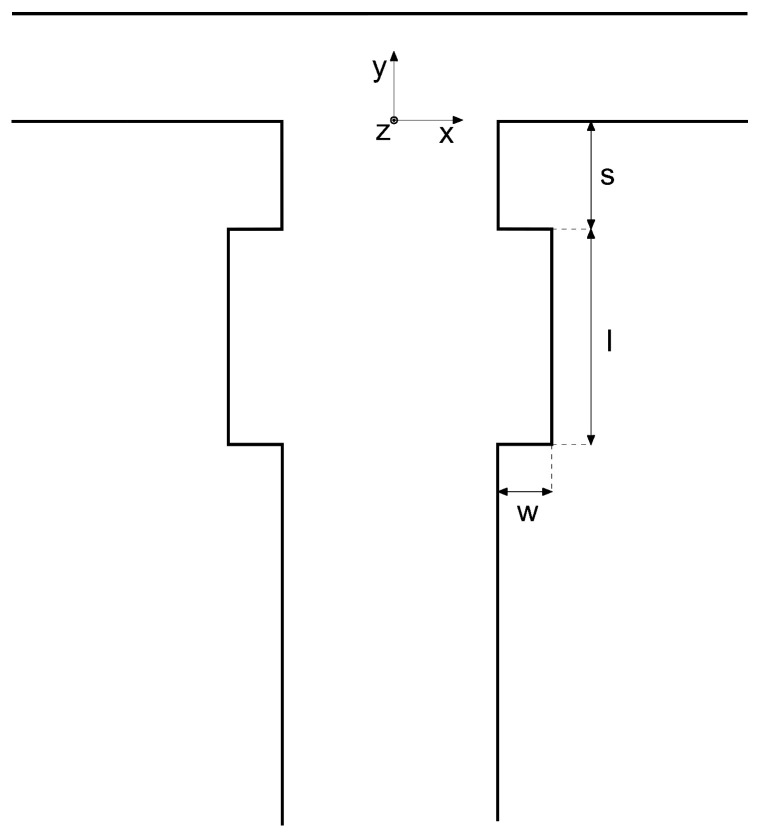
Sketch of the geometry.

The inlet channels of the T-shaped micro-junction have a width of 1 mm, while the outlet channel (also called “mixing channel” in the following) is 2 mm wide. The inlet channel length is 40 mm, and the mixing channel is 60 mm long, enough to provide a fully developed flow at the confluence and a complete development of the vortical structures, respectively. The height of the microchannels is 1 mm, as in the work of Mariotti et al. [31], the hydraulic diameter is *d* = 1.33 mm, and the reference system is placed at the bottom center of the beginning of the mixing channel (see Figure 1).

A sketch of the rectangular cavities is presented in Figure 1. The cavity geometry is defined by a set of three parameters: the distance from the confluence region of the beginning of the cavity, *s*, the cavity total length, *l*, and its width, *w*. In Table 1, all the combinations of these geometrical parameters considered in this work are summarized. Parameters are made non-dimensional through the hydraulic diameter *d*, i.e., S=s/d, W=w/d, and L=l/d.

Cavity parameters for the case TJC are chosen based on preliminary studies (not reported herein for the sake of brevity) and are motivated by two requirements: (i) we want to place the cavity just downstream of the confluence region, and (ii) the cavity should be small compared with the mixing channel characteristic size.

In Section 3.3, the effect of small variations of these parameters will be investigated. Indeed, the cases *S*, *W*, and *L* (see Table 1) refer to the robustness analysis of the configuration TJC with respect to the parameters *s*, *w*, and *l*, respectively.

### 2.2. Solver Settings and Grid

Assume that the two incoming fluids consist of pure water on one side and an aqueous solution of a miscible solute (e.g., a dye) on the other. As the solution is very dilute, the physical properties of the fluid are uniform and coincide with those of water. As such, the fluid is incompressible and the mixing process takes place at isothermal conditions so that the governing equations consist of the Navier–Stokes equation and the transport equation for the dilute solute.

In non-dimensional terms, these equations read:

(1) $$\begin{array}{c} \nabla \;\cdot \;u=0, \end{array}$$

(2) $$\begin{array}{c} \frac{\partial u}{\partial \theta }+\left(u\;\cdot \;\nabla \right)u=-\nabla P+\frac{1}{Re}\nabla^{2}u, \end{array}$$

(3) $$\begin{array}{c} \frac{\partial \phi }{\partial \theta }+u\;\cdot \;\nabla \phi =\frac{1}{Pe}\nabla^{2}\phi . \end{array}$$

Here, time, length, and velocities are normalized with the convective time d/U, the hydraulic diameter *d*, and the inlet bulk velocity *U*, respectively. **u** is the non-dimensional velocity vector, *P* is the modified non-dimensional pressure, and ϕ is the mass fraction of the passive scalar.

The non-dimensional numbers are the Reynolds number Re=Ud/ν, and the Peclet number Pe=Ud/D. As for the fluid properties, ν is the kinematic viscosity of water (i.e., $\nu =10^{-6}$ m^2^/s)—defined as the ratio of the dynamic viscosity μ and the density ρ—while D is the diffusion coefficient of the scalar (i.e., $D=3\;\cdot \;10^{-10}$ m^2^/s). As the Schmidt number $Sc=\nu /D\approx 3\;\cdot \;10^{3}$ is very large, mass diffusion is very slow compared to momentum transport, therefore the dilute solute can be described as a passive tracer (see discussion in [31,61]).

The software ANSYS Fluent [81], based on a finite-volume formulation, is used to carry out the numerical simulations. Steady-state and transient simulations are performed, depending on the considered flow regime. As for the time discretization, a second-order implicit scheme is used with a time step corresponding to a CFL number ≅5. Convergence criteria are the same as in [31,43,44] with no cavities. As boundary conditions, no-slip velocity was imposed at the channel walls, a uniform velocity was set at the inlet sections (each inlet fluid enters with the same velocity), and a pressure outlet was established at the ambient condition at the end of the mixing channel. The simulations were all initialized using uniform conditions to avoid any kind of hysteresis.

The numerical grid is generated according to the grid independency studies carried out in [31,43,44,82], in which the T-shaped micro-junction without cavities was studied. The number of elements in the inlet-channel and outlet-channel cross-sections are 40 × 40 and 40 × 80, respectively [31]. Hence, the characteristic cell size corresponds to 25 μm. Specifically, the cells are cubical at the confluence regions and elongate towards the inlets and the outlet. Inside the cavity, the computational cells have the same dimensions as in the mixing channel cross-section.

### 2.3. Evaluation of Mixing Performance

The mixing performance is evaluated in terms of the degree of mixing ($\delta_{m}$) [32,83]. Recalling that the flow variance evaluated at a given cross-section (*S*) of the mixing channel is:

(4) $$\sigma^{2}=\frac{1}{A}\int_{S}{\left(\phi -\bar{\phi }\right)}^{2}u_{y}dS,$$

where *A* is the extension of *S*, $\bar{\phi }$ is the mass fraction of the passive scalar in case of complete mixing ($\bar{\phi }=0.5$), and $u_{y}$ is the *y*-velocity. Thus, we define the degree of mixing [32,83] as:

(5) $$\delta_{m}=1-\frac{\sigma }{\sigma_{max}},\;\sigma_{max}=\sqrt{\left(1-\bar{\phi }\right)\bar{\phi }},$$

where $\sigma_{max}$ is the maximum of the flow variance (σ) obtained when we consider complete segregation of fluids. $\delta_{m}$ is 0 in case of two segregated streams, or 1 when complete mixing is achieved.

## 3. Results

### 3.1. Flow Control and Device Performance

**Figure 2:**
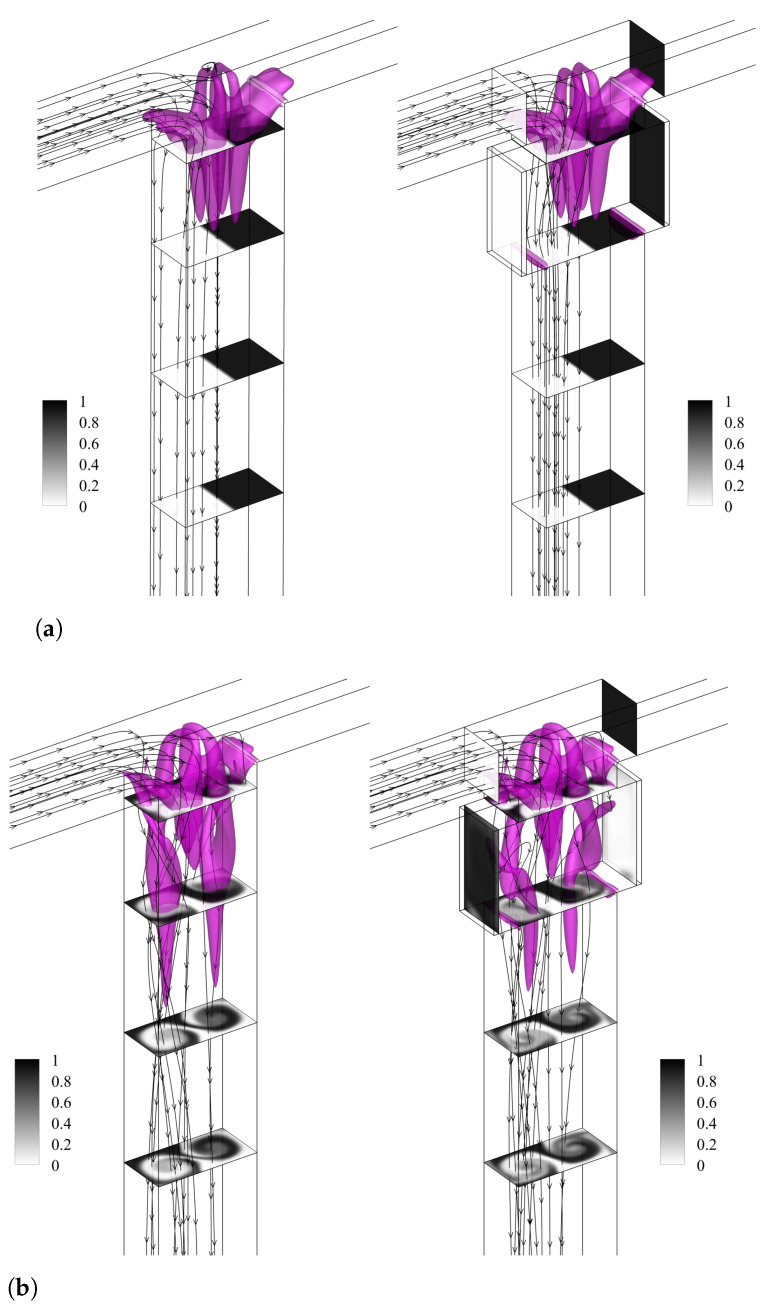

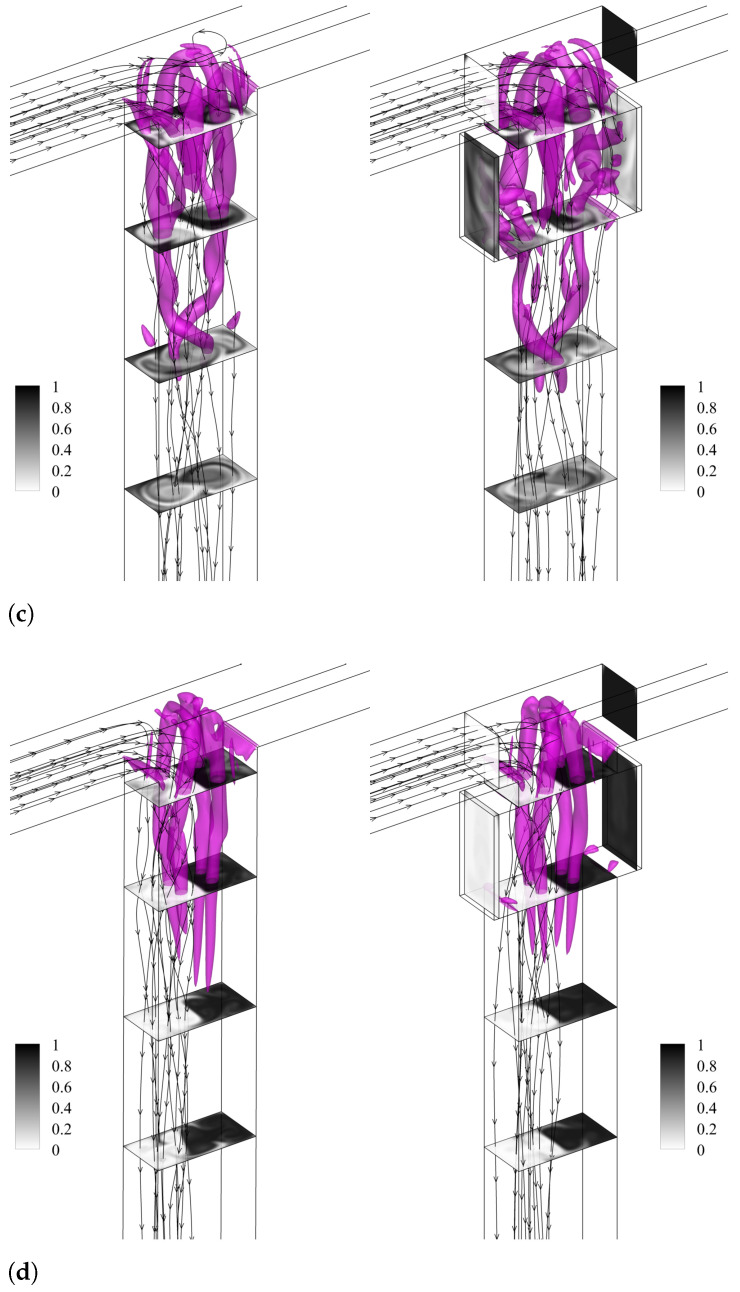
Isosurfaces of the $\lambda_{2}$ vortex-indicator and contours of non-dimensional dye concentration at the Y=−0.375,−1.875,−3.375, and −4.874 cross-sections along the mixing channel for TJ case (left panel) and TJC case (right panel). Considered Reynolds numbers: (**a**) Re=100, (**b**) Re=200, (**c**) Re=300, and (**d**) Re=400.

The flow patterns for TJ and TJC cases are compared in Figure 2 for Reynolds numbers Re = 100, 200, 300, and 400. The passive-scalar concentration field along different cross-sections in the mixer geometry, the flow streamlines, and the vortical structures identified through the isocontour of the vortex indicator $\lambda_{2}$ [84] are shown for the TJ case (left panel) and for the TJC case (right panel).

At Re=100 (Figure 2a), the two streams remain segregated along the mixing channels in both geometries. Two pairs of symmetric U-shaped vortical structures develop in the confluence region, leading to the formation of four counter-rotating vortical structures in the mixing channel, characteristic of the so-called vortex regime [31]. The double-mirror symmetry is preserved, leading to a poor-mixed regime in the TJ configuration. The presence of cavities does not alter the fluid motion and the symmetric flow pattern, seen in the TJ case. Furthermore, we observe that each cavity is filled by the fluid stream coming from the same inlet channel side, as the streamlines (represented in the right panel in Figure 2a) do not cross over the channel centreline.

Figure 2b shows the engulfment regime at Re=200 in a TJ. Differently from the vortex regime, the two U-shaped vortices are tilted in the confluence region. The symmetry breaking promotes only the strongest vortical leg for each U-shaped vortex to be present in the mixing channel (for more details see [31]). The two co-rotating vortices develop intense convection in the microchannel. Nearby the confluence, each inlet fluid moves toward the opposite side of the mixing channel wall, as highlighted by the streamlines in the left panel of Figure 2b, and an asymmetric fluid pattern is formed, clearly shown by the cross-sectional contours of the passive scalar.

Moving to the right panel of Figure 2b, the presence of the cavities does not interfere with the formation of the vortical structures in the engulfment regime. Moreover, we observe that each cavity is filled with fluid coming from the opposite side. This aspect is fundamental to improve the mixing of the fluid streams in the region near the mixing-channel lateral walls, which has been identified in Mariotti et al. [31] as the one characterized by a low level of mixing (the two co-rotating vortices are placed in the center of the mixing-channel cross-section). Indeed, comparing the cross-sectional contours between the left and right panels of Figure 2b, we can identify a wider gray area for the T-shaped micro-junction having cavities on the lateral channel walls.

The periodic asymmetric regime at Re=300 is shown in Figure 2c for the TJ and TJC cases, by using the same instants in the temporal cycle. This regime is characterized by the periodic shedding of a vorticity blob (see [32] for details).

Again, the introduction of cavities does not alter the vortical structures present in the mixing channel. As for Re=200, the cavities contain streams coming from the opposite inlet side, further promoting mixing near the channel walls. The same holds for all the time instants of the periodic cycle (not shown here for the sake of brevity).

In the periodic symmetric regime, the flow streams are segregated with a periodic motion of the top parts of mainly-symmetric vortical structures [32]. Figure 2d shows an instant of the periodic cycle. As found for the vortex regime, the streamlines do not cross over the centreline of the mixing channel, and the fluid pattern is symmetric. Thus, the cavities (right panel in Figure 2d) are filled with the stream coming from the inlet channel placed at the same side, without a beneficial effect on the mixing degree.

**Figure 3:**
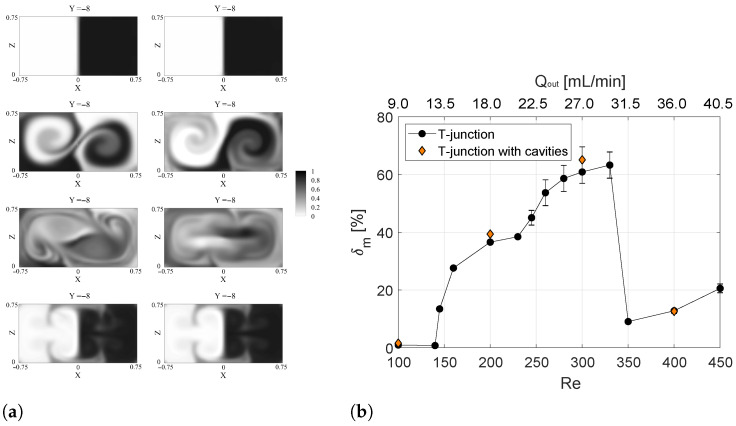
(**a**) Contours of non-dimensional dye concentration at the Y=−8 cross-section along the mixing channel for the TJ case (left panel) and the TJC case (right panel) from top to bottom: Re=100, Re=200, Re=300, and Re=400. (**b**) Degree of mixing evaluated at the Y=−8 cross-section as a function of Reynolds number and of the volumetric flow rate of water ($Q_{out}$) in the outlet channel.

A quantitative evaluation of the effect of the cavity on the degree of mixing, $\delta_{m}$, is summarized in Figure 3b. The degree of mixing is evaluated at the Y=−8 cross-section in the mixing channel for TJ and TJC cases (TJ results are from [32]). In Figure 3b, the results for the unsteady regimes are indicated in terms of mean values (symbols), while bars indicate the range of $\delta_{m}$ values spanned in the cycle. The dye concentration pattern for TJ and TJC cases are compared in Figure 3a. It is evident from the latter figure that the improvement of the mixing is achieved using a pair of cavities, both in the steady engulfment and the periodic asymmetric regimes. Mixing is promoted mainly near the walls, leading to an increase in the degree of mixing of the TJC case with respect to the TJ one corresponding to 7.63% and 6.90% for Re=200 and Re=300, respectively. On the other hand, no significant improvements are detected in $\delta_{m}$ at Re=100 and Re=400.

It should also be noted that the cost of mixing, evaluated in terms of Pressure Drops (PD), does not change when the pair of cavities is introduced in the mixing channel, as can be derived from Table 2. Pressure drops are evaluated between the X=2 section of the inlet channel and Y=−18 section of the mixing channel (where the vortical structures are formed), and they are made non-dimensional by dividing by $\rho U^{2}$. We may observe that the differences in PD between the TJ and the TJC are negligible for all the considered operating conditions.

### 3.2. Effect of Cavities on the Mixing Mechanism in the Steady Engulfment Regime

**Figure 4:**
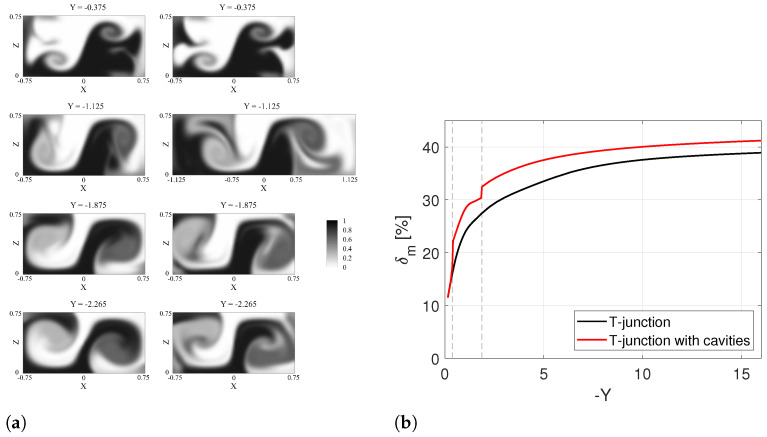
(**a**) Contours of non-dimensional dye concentration at the Y=−0.375,−1.125,−1.875, and −2.265 cross-sections along the outlet channel for the TJ case (left panel) and the TJC case (right panel) in numerical simulations at Re=200. (**b**) Degree of mixing of the T-junction as a function of *Y* at Re=200.

To gain a deeper insight into the physical mechanisms through which the introduction of the cavities causes a further increase in the mixing performance in the engulfment regime (Re=200), the variation of the mixing degree along the mixing channel for TJ and TJC cases is evaluated as shown in Figure 4. In the same Figure, the passive scalar fields in different TJ and TJC cross-sections upstream, within, and downstream the cavity region are compared.

Upstream the cavity region, i.e., at Y=−0.375 (top panel of Figure 4a), no significant differences between the two fields are found, as confirmed by the same value of the mixing degree reported in Figure 4b at this coordinate.

In the cavity region (second panel from the top in Figure 4a), it is evident that the black stream entering from the right is remarkably present in the left cavity, and vice versa for the white stream, with a consequent sudden increase in $\delta_{m}$ in Figure 4b at the coordinate corresponding to the beginning of the cavity.

Further downstream (see, e.g, the Y=−1.875 and Y=−2.265 cross-sections in Figure 4a), mixing of the TJC case is always above the one for the TJ case, as mixing is enhanced near the lateral walls of the mixing channel.

Summarizing, three different regions can be defined for the behavior of $\delta_{m}$ along the mixing channel and highlighted with dashed lines in Figure 4b:

- the *confluence zone* (−0.375<Y<0), wherein both geometries’ similar mixing performances are present;
- the *cavity zone* (−1.875<Y<−0.375) (represented by the dashed lines in Figure 4b), where the presence of cavities further improves the mixing between the two inlet streams ($\delta_{m}\left(Y=-1.875\right)$ = 32.5%) compared to the TJ case ($\delta_{m}\left(Y=-1.875\right)$ = 27.5%);
- the *flow zone* (Y<−1.875) where the two curves for TJ and TJC increase in parallel.

**Figure 5:**
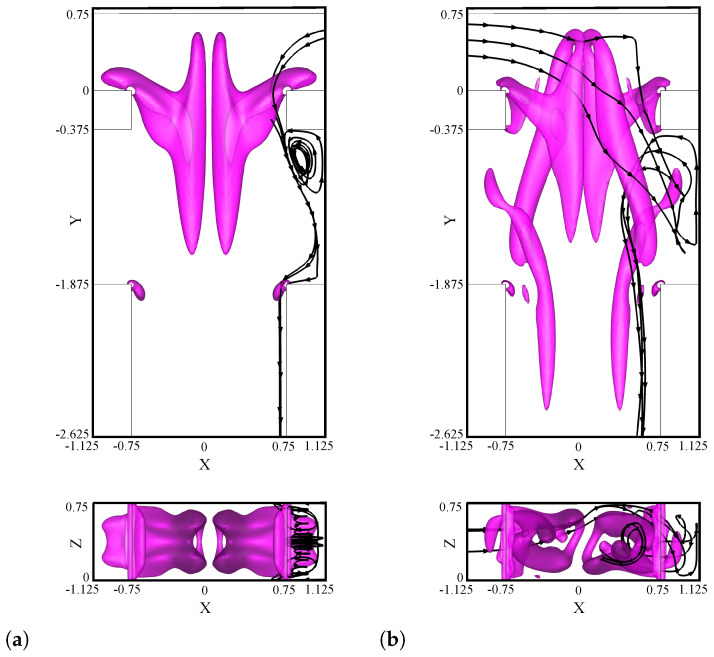
Top view and outlet view of the isosurfaces of the $\lambda_{2}$ vortex-indicator and streamlines inside the right cavity at (**a**) Re=100 and (**b**) Re=200.

We focus now on the flow pattern inside the cavities by comparing the results at Re=100 (Figure 5a) and Re=200 (Figure 5b). From the analysis of the flow streamlines, it is evident that at Re=200 the inlet fluid coming from the left crosses the channel and moves into the right cavity thanks to characteristic vortices of the engulfment regime. Afterward, the streamlines enter and recirculate inside the cavity to exit into the mixing channel at a different position.

Hence, we may gather that the cavity further enhances the mixing by trapping the fluid coming from the other stream in the recirculation near the wall, which reaches the opposite wall thanks to the convection induced by the vortical structures in the mixing channel. This fluid motion does not happen in the segregated regimes (see e.g., Re=100 in Figure 5a) because the fluid entering from one side is not able to reach the opposite cavity.

### 3.3. Robustness to the Cavity Parameters in the Engulfment Regime

In this section, the effects of some cavity modifications are investigated to ascertain whether the performance of this passive flow-control device is robust with respect to small variations of the cavity geometry from the standard TJC case. To this aim, we computed the degree of mixing at Y=−8 for all the configurations listed in Table 1. We carried out CFD simulations on twelve additional geometries of cavities, changing only one parameter at a time and keeping the others as in the TJC case.

**Figure 6:**
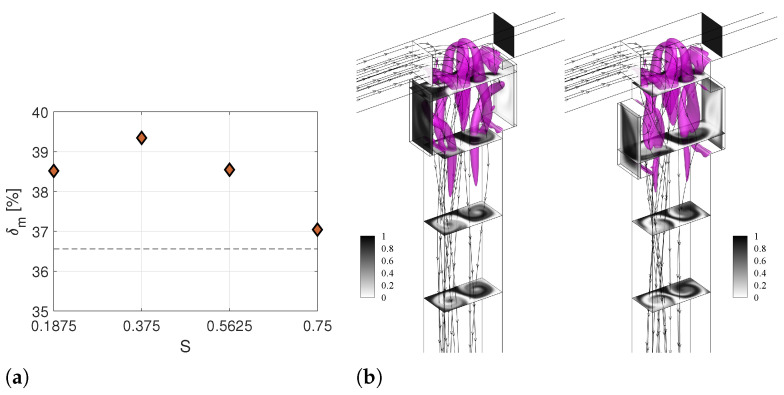
(**a**) Degree of mixing at the Y=−8 cross-section of the T-microchannel with cavities located at different distance (*S*) from the junction. (**b**) Isosurfaces of the $\lambda_{2}$ vortex-indicator and contours of non-dimensional dye concentration at the Y=0.375,−1.875,−3.375, and −4.874 cross-sections along the outlet channel for geometries with the shortest (left panel) and longest (right panel) distance *S*, i.e., Case S.1 S=0.1875 and Case S.3 S=0.75, respectively, at Re=200.

Figure 6a shows the values of $\delta_{m}$ changing the non-dimensional distance from the confluence *S*. The horizontal dashed line represents the performance of the TJ case that is equal to $\delta_{m}$ = 36.56% [31]. For all the considered values of *S*, the mixing efficiency is larger than the one of the mixer without cavities. In particular, the TJC is the best configuration for mixing reaching $\delta_{m}$ = 39.35%. The flow pattern for the smaller and higher values of *S* (S.1 and S.2 cases) are shown in the left and right panels of Figure 6b, respectively. The flow field for case S.1 is very similar to the one found for the TJC case. On the other hand, increasing the distance from the confluence region may hamper the crossing of flow streamlines to the opposite cavity, leading to a decrease in the effectiveness of the cavities (see right panel Figure 6).

**Figure 7:**
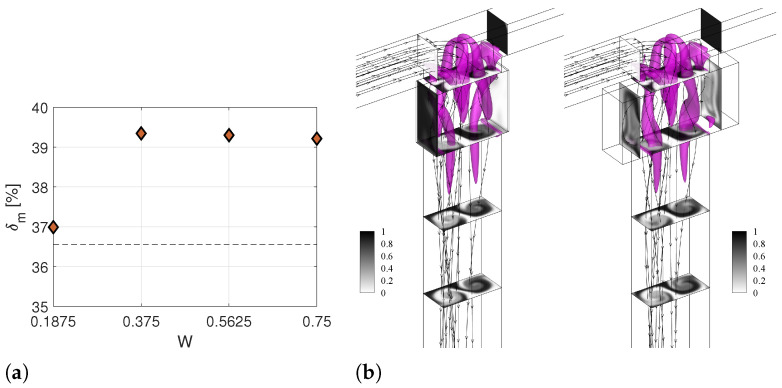
(**a**) Degree of mixing of the T-microchannel with different cavity width (*W*) at Y=−8. (**b**) Isosurfaces of the $\lambda_{2}$ vortex-indicator and contours of non-dimensional dye concentration at the Y=0.375,−1.875,−3.375, and −4.874 cross-sections along the outlet channel for geometries with the shortest (left panel) and longest (right panel) length *W*, i.e., Case W.1 W=0.1875 and Case W.3 W=0.75, respectively, at Re=200.

Increasing the cavity width above the TJC value W=0.375 does not significantly modify the mixing performance, as the degree of mixing is almost constant for cases W.2 and W.3 (Figure 7a). Nevertheless, it is worth noting that using an excessively wide cavity might promote the presence of fluid dead zones, which is not desirable for mixing and reaction processes. For the smallest value of *W*, i.e., W=1.875, cavities are not effective. It is reasonable to infer that a minimum value of the cavity depth exists to have a recirculation of flow inside the cavity. Below this value, the mixing performance is practically the one for the T-junction without the cavities. Indeed, the flow pattern for W.1 is the same as for the TJ case (compare the left panel in Figure 7b and the right panel in Figure 2b).

**Figure 8:**
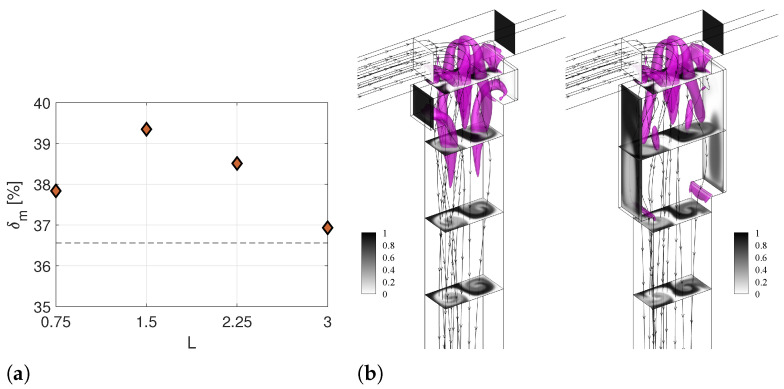
(**a**) Degree of mixing of the T-microchannel with different cavity length (*L*) at Y=−8. (**b**) Isosurfaces of the $\lambda_{2}$ vortex-indicator and contours of non-dimensional dye concentration at the Y=0.375,−1.875,−3.375, and −4.874 cross-sections along the outlet channel for geometries with the shortest (left panel) and longest (right panel) length *L*, i.e., Case L.1 L=0.75 and Case L.3 L=3.00, respectively, at Re=200.

Finally, the effect of the cavity length *L* is analyzed in Figure 8. Again, for all the considered values of *L*, a higher degree of mixing is found compared to the TJ case (dashed lines in Figure 8a). Observing Figure 8b, the design with the lowest *L* has a vortical structure similar to the cases shown for the TJC case in the right panel of Figure 2b. However, the smallest extension of the cavity along the mixing channel hampers the effect of the cavity introduction. On the contrary, when long cavities are exploited, the vortical structures characteristic of the engulfment regime seem to lose intensity, and the middle area of the cross-sectional contours looks more segregated than in the TJC case.

## 4. Discussion and Future Perspectives

In this work, a particular T-mixer configuration, in which the mixing channel cross-section is equal to two times the inlet channel cross-sections, is considered to assess the flow control device performance and the robustness to changes in cavity geometry. However, the width of the mixing channel (compared with the one of the inlet channels) is a crucial parameter for the onset of the different flow regimes occurring in the T-mixer by increasing the Reynolds number (see, e.g., [34,35,82]). In particular, the onset of the engulfment regime initiates at lower Reynolds numbers if the flow decelerates in the mixing channel (i.e., the mixing channel is more than twice as wide as the inlet channels). It is reasonable to infer that the physical mechanism triggering mixing with cavities and the interplay between the recirculation embedded in the cavity region and the vortical structures formed in the different flow regimes of the T-shaped micro-junction still holds also for different mixing channel cross-sections. Nevertheless, an optimization of the cavity parameters is needed for each considered T-mixer geometry to maximize the flow control device performance.

More challenging is the comparison between the effectiveness of this flow control strategy and the other strategies—mentioned in the Introduction—to modify the T-geometry and intensify the mixing. Even though higher increases in the mixing degree can be obtained with the latter methods, it is evident how they massively impact the change of the mixer geometry. As an example, multiple cavities were successfully adopted to reduce the mixing length in convergent-divergent micromixers [63,66,69] and multiple cavities in a staggered configuration are also effective to improve the efficiency of T-shaped mixers improving the fluid exchange between the two streams [64,68]. Nevertheless, these configurations promote a higher increase in the pressure drops and, thus, of the mixing cost, and a higher complexity of the manufacturing process.

Finally, the present paper has considered water as a working fluid and equal and constant velocities of the streams in the inlet channels. In the case of different inlet conditions, the mass exchange should be considered in the evaluation of the cavity performance. For instance, Ottino and coworkers [85,86,87] found that convection-enhanced transport can be accomplished in open cavities in the case of inlet periodic flow conditions thanks to the formation of lobes in the cavity flow. Moreover, for a dilute suspension of particles, Haddadi and Di Carlo [79] observed the development of a spiralling flow, which induced the exchange of fluid mass between the main channel flow and the vortical flow in the cavity. Furthermore, a systematic evaluation of the cavity performance should be carried out for these cases in future research activities.

## 5. Conclusions

The effect of the introduction of a pair of symmetric cavities in the foremost part of the mixing channel of a T-shaped micro-junction has been investigated through numerical simulations in flow regimes occurring by increasing the Reynolds number. The introduction of cavities leads to an increase in mixing in the steady engulfment and in the periodic asymmetric regimes. Such an intensification of mixing does not require additional energy, because pressure drops do not change.

The analysis of the vortical structures and of the flow streamlines provides insight into fluid and mixing mechanisms occurring inside the cavities during the steady engulfment regime. In this regime, the fluid stream entering on one side reaches the opposite wall in the confluence region, thanks to the convection induced by the vortical structures. This fluid is trapped for a while in the recirculation region present inside the cavity and this further enhances mixing, especially near the wall.

Robustness analysis of the flow-control device showed that the mixing improvements also remain significant for small changes of the cavity shape parameters and position around the optimum ones. Moreover, a minimum value has been identified for the cavity width, i.e., W=0.375. Below this value, the effect of cavity introduction is negligible for the considered width, aspect ratio, and configuration of the microchannel.

Summarizing, the introduction of one pair of cavities enhances the mixing efficiency while preserving the simple geometry of the T-junction and without significant additional pressure drops. Future works should be aimed at expanding the investigation to a wider range of cavity shapes and operational conditions. In addition, cavities might be exploited to enhance the mixing in other T-geometries and different micromixer shapes, e.g., arrow or cross-shaped mixers.

## Figures and Tables

**Table 1:** Geometrical dimension of the cavities used in the simulations.

Case	*L*	*W*	*S*
TJC	1.50	0.375	0.375
S.1	1.50	0.375	0.1875
S.2	1.50	0.375	0.5625
S.3	1.50	0.375	0.75
W.1	1.50	0.1875	0.375
W.2	1.50	0.5625	0.375
W.3	1.50	0.75	0.375
L.1	0.75	0.375	0.375
L.2	2.25	0.375	0.375
L.3	3.00	0.375	0.375

**Table 2:** Non-dimensional pressure drops in TJ and TJC cases.

	*Re* = 100	*Re* = 200	*Re* = 300	*Re* = 400
TJ case	0.633	0.761	0.982	1.01
TJC case	0.629	0.763	0.991	1.01
difference	0.63%	−0.26%	−0.92%	0.20%

## Data Availability

Supported data are not reported.
